# Development of a sensitive molecular diagnostic assay for detecting *Borrelia burgdorferi* DNA from the blood of Lyme disease patients by digital PCR

**DOI:** 10.1371/journal.pone.0235372

**Published:** 2020-11-30

**Authors:** Srirupa Das, Denise Hammond-McKibben, Donna Guralski, Sandra Lobo, Paul N. Fiedler

**Affiliations:** 1 Department of Pathology Research, Nuvance Health, Danbury, CT, United States of America; 2 Rudy L. Ruggles Biomedical Research Institute, Nuvance Health, Danbury, CT, United States of America; Universita degli Studi di Parma, ITALY

## Abstract

Lyme disease patients would greatly benefit from a timely, sensitive, and specific molecular diagnostic test that can detect the causal agent *Borrelia burgdorferi* at the onset of symptoms. Currently available diagnostic methods recommended by the Centers for Disease Control and Prevention for Lyme disease involve indirect serological tests that rely on the detection of a host-antibody response, which often takes more than three weeks to develop. With this process, many positive cases are not detected within a timely manner, preventing a complete cure. In this study, we have developed a digital polymerase chain reaction (PCR) assay that detects Lyme disease on clinical presentation with a sensitivity two-fold higher than that of the currently available diagnostic methods, using a cohort of patient samples collected from the Lyme disease endemic state of Connecticut, USA, in 2016–2018. Digital PCR technology was chosen as it is more advanced and sensitive than other PCR techniques in detecting rare targets. The analytical detection sensitivity of this diagnostic assay is approximately three genome copies of *B*. *burgdorferi*. The paucity of spirochetes in the bloodstream of Lyme disease patients has hindered the clinical adoption of PCR-based diagnostic tests. However, this drawback was overcome by using a comparatively larger sample volume, applying pre-analytical processing to the blood samples, and implementing a pre-amplification step to enrich for *B*. *burgdorferi*-specific gene targets before the patient samples are analyzed via digital PCR technology. Pre-analytical processing of blood samples from acute patients revealed that the best sample type for Lyme disease detection is platelet-rich plasma rather than whole blood. If detected in a timely manner, Lyme disease can be completely cured, thus limiting antibiotic overuse and associated morbidities.

## Introduction

Lyme disease (LD), a systemic tick-borne infection caused by the bacteria *Borrelia burgdorferi*, is the most common vector-borne disease in the USA. According to the Centers for Disease Control and Prevention (CDC), an estimated 300,000 new cases of LD occur in the USA every year. However, only 10% of these cases are actually reported and diagnosed [1]. Due to the non-specific flu-like symptoms of LD and a lack of reliable testing for the early stages of infection, diagnosis is very challenging. According to the CDC, the characteristic symptom of LD, i.e., a typical bulls-eye rash known as erythema migrans (EM) develops in only 70%–80% of patients and can often be confused with other similar rashes [2]. The current CDC-approved diagnostic method for LD detection is serological two-tiered testing (TT testing), which includes a screening test by an enzyme-linked immunosorbent assay (ELISA) and a specificity test by western blot. This test is inaccurate in the early stages of disease, as it relies on the indirect detection of a host-antibody response that often takes three weeks or more to develop. As a result, 25%–50% of positive LD cases are missed during initial diagnosis. Because the test is not likely to be positive until 3–6 weeks post-infection, the CDC recommends that doctors who suspect LD based on symptoms and epidemiological information prescribe antibiotics even if the test is negative [3]. Early diagnosis is critical for minimizing the long-term effects and morbidity associated with LD and for ensuring a complete cure.

Thus far, diagnostic LD detection methods that involve laboratory culturing or polymerase chain reaction (PCR) have not been successful. While *B*. *burgdorferi* is recalcitrant to culturing under laboratory conditions, a clinically relevant PCR assay for LD detection from blood has not been established due to the insufficient sensitivity of conventional PCR methods and the extremely low levels of spirochetes found in the blood of infected patients [4–7]. In the past, PCR methods aiming to detect *Borrelia* infection using blood of acute LD patients suffered from low sensitivities of approximately 20% [8,9]. As a result, these assays are unreliable for LD detection in clinical blood samples. The PCR results are also discordant depending on the type of specimen tested and the symptoms reported by the patient, limiting the clinical adoption of PCR testing for LD [4–7]. Prior culturing of *B*. *burgdorferi* under laboratory conditions from patient samples followed by PCR has resulted in better detection rates, indicating an unusually low bacterial load in humans [10]. Current advances in molecular techniques have led to improved DNA extraction and amplification techniques, resulting in the detection of low copy numbers of *Borrelia* DNA from larger patient sample volumes [11]. Newer PCR techniques in LD detection, such as real-time quantitative PCR (qPCR) and nested PCR, have demonstrated improved sensitivity for detecting *B*. *burgdorferi* [10,12,13]. Due to the low bacterial loads of spirochetes in the circulating blood of infected humans, investigators have utilized larger volumes of patient blood to boost the LD detection rate [7]. The development of a highly sensitive diagnostic test to directly detect *B*. *burgdorferi* in blood would significantly enhance the detection of LD in early stages, when treatment is most effective.

In this study, we have used digital PCR (dPCR) to develop a sensitive method for directly detecting LD at clinical presentation. dPCR is a quantitative PCR method that sensitively and reproducibly measures the amount of DNA or RNA present in a sample. In dPCR, samples are partitioned into individual wells such that each well receives either one target or zero targets prior to PCR amplification. Absolute quantitation of the target is achieved by counting the number of positive versus negative reactions using Poisson statistics [14]. Compared with other PCR methods, the partitioning of samples during dPCR leads to a significantly improved sensitivity for detecting rare alleles, low pathogen loads, and targets in limited clinical samples [15]. To further improve LD detection in patients, we have also incorporated pre-analytical processing of blood samples and a pre-amplification step to enrich for *B*. *burgdorferi*-specific target DNA, prior to sample analysis by dPCR. The assay reported herein can detect LD at twice the sensitivity of the current CDC-recommended diagnostic methods.

## Materials and methods

### Ethics statement

This study was approved by the Biomedical Research Alliance of the New York Institutional Review Board (IRB# 16–104 and 17–202). Participants provided written informed consent prior to inclusion in the study.

### Culture of a *B*. *burgdorferi* strain

*Borrelia burgdorferi* strain B31 was purchased from the American Type Culture Collection (ATCC), Manassas, Virginia (Catalog No. #35210), and was maintained in complete BSK-H medium containing 6% rabbit serum (complete medium from Sigma-Aldrich, St. Louis, Missouri) at 33°C.

### Collection of LD patient samples

Paired whole blood (WB) and serum samples were collected from 46 clinically diagnosed LD patients during 2016–2018 from an LD-endemic area (Connecticut, USA). During the course of the study, seven of these patients dropped out after the initial visit, and three patients dropped out after the second visit. Samples were collected from each patient at the initial pre-treatment (acute), during treatment (2 weeks post-diagnosis), and post-treatment stages (6 weeks post-diagnosis). Patients were referred to the study by their primary care provider immediately following their LD diagnosis. Patients included in the study most often presented with a rash consistent with EM, a known tick bite, fever, and other symptoms consistent with *B*. *burgdorferi* infection. Patients who had a known history of LD during the past 5 years, who were pregnant, or who had been taking antibiotics for more than 72 h were excluded from the study.

WB samples from patients were collected in Cyto-Chex® BCT tubes (Streck, La Vista, Nebraska) and a Vacutainer serum-separator tube (BD Biosciences, San Jose, California). Non-LD controls were collected from the state of Connecticut, USA (100 samples), under our approved IRB and from Tennessee, USA (30 samples purchased from Tennessee Blood Services, Memphis, Tennessee). The non-LD controls were chosen from people who had never been diagnosed with LD or had not been diagnosed within the last 5 years prior to the study. The control patients had no ongoing symptoms associated with the disease. We also obtained de-identified blood samples from clinically diagnosed LD patients with positive IgM western blot results from Danbury Hospital, Danbury, Connecticut, under our approved IRB protocol for assay optimization.

### Serological analysis

Serum samples were subjected to TT testing for *B*. *burgdorferi* at Danbury Hospital, following the CDC-recommended guidelines [16]. Serum samples were also sent to the Mayo Clinic, Rochester, Minnesota, for C6 peptide Lyme ELISA testing. The TT testing results (**S1 Table**) were used to evaluate the efficiency of the LD PCR diagnostic assay developed herein.

### Pre-analytical processing of blood samples

WB samples were subjected to pre-analytical processing prior to DNA extraction. The blood samples were processed to enable their separation as serum, plasma, platelet-rich plasma (PRP), and WB for each patient. All samples were aliquoted at 1 mL per cryovial and stored at -80°C before use. For serum collection, a Vacutainer SST tube was centrifuged at 1000 g for 10 min at 4°C, and the supernatant was collected. Plasma was collected by centrifuging the WB sample at 1200 g for 10 min at 4°C, and the supernatant was stored. For PRP collection, WB was centrifuged at 260 g for 10 min at room temperature (RT), and the supernatant (above the buffy coat) was collected. However, the PRP from the 2017 patient samples was processed differently to evaluate the efficiency of different pre-analytical processing and storage methods. In 2017, the PRP was pelleted down by another round of centrifugation at 15,000 rpm for 10 min at 4°C. The supernatant was discarded, and the PRP pellet was stored at -80°C until further use.

### DNA extraction and precipitation

DNA extraction was performed from 1 mL of the different sample types using a QIAamp DNA mini kit (Qiagen, Hilden, Germany) as per the vendor’s instructions with slight modifications. All samples were pelleted down at 15,000 rpm for 10 min at 4°C, and the supernatant was discarded. The pellets underwent bacterial DNA isolation via resuspension in 180 μL of Buffer ATL and 20 μL of proteinase K. The lysate was incubated for 1 h at 56°C in a thermomixer (Eppendorf, Hamburg, Germany) with shaking. Subsequently, 200 μL of Buffer AL and 10 μg of poly(A) carrier DNA (Roche, Basel, Switzerland) were added to the lysate, which was incubated for an additional 10 min at 70°C. Next, 230 μL of molecular-biology-grade ethanol (Sigma-Aldrich, St. Louis, Missouri) was added to the lysate, which was then passed through a QIAamp mini spin column at 6000 g for 1 min at RT. The filtrate was discarded, and the spin column was washed with wash buffers AW1 and AW2, as per the vendor’s instructions. Finally, DNA was eluted twice with 150 μL of Buffer AE (pre-heated to 65°C), resulting in a total volume of ~300 μL. Bacterial DNA extraction from WB was conducted using a QIAamp DNA blood mini kit (Qiagen, Hilden, Germany) following the vendor’s recommended protocols.

### Processing of extracted DNA

The total volume of extracted DNA was precipitated with 1/10^th^ volume of 3M sodium acetate (Invitrogen, Carlsbad, California) and a double volume of chilled molecular-biology-grade ethanol (Sigma-Aldrich, St. Louis, Missouri) at -20°C overnight. The precipitated DNA was pelleted down at 15,000 rpm for 10 min at 4°C. The supernatant was discarded, and the pellet was tapped, dislodged, and washed twice with 700 μL of 70% ethanol, followed by centrifugation at 15,000 rpm for 10 min at 4°C. The clean DNA pellet was dried in a sterile environment at 37°C for 1 h or until dry. The DNA pellet was dissolved in 3.125 μL/12.5 μL of DNA suspension buffer (Teknova, Hollister, California) as needed. The re-suspended DNA was stored at -20°C until further use.

### *B*. *burgdorferi*-specific TaqMan assays

Bioinformatic tools (Basic Local Alignment Search Tool [BLAST] and Primer BLAST [NCBI]; Multiple Sequence Alignment by CLUSTALW) were used to identify four unique *B*. *burgdorferi*-specific gene sequences to enable custom manufacture of TaqMan assays (**Table 1**) by Life Technologies (Carlsbad, California). *B*. *burgdorferi* strain B31 was the source of the sequences chosen from the four different genes: *ospA* (GenBank: AE000790.2), *ospC* (GenBank: U01894.1), *fla* (GenBank: NC_001318.1), and *rpoB* (GenBank: AE000783.1).

**Table 1:** Primer and probe sequences of the TaqMan assays.

Gene	Forward Primer (5’-3’)	Reverse Primer (5’-3’)	Probe (5’-3’)	Fluorophore-Quencher
*ospA*	GGCACTTCAACTTTAACAATTACTGTAA	GCCATTTGAGTCGTATTGTTGTACTGTA	ACACAAGGTCTTTAGTTTTT	FAM-MGB/NFQ
*ospC*	GGTTGAAGCGTTGCTGTCATCTATA	TCGGTATCCAAACCATTATTTTGGTGTA	CTTTAGCAGCAATTTC	FAM-MGB/NFQ
*fla*	TCTAGTGGGTACAGAATTAATCGAGCTT	GAGCATTAATCTTACCAGAAACTCCCA	CCAGCAGCATCATCAG	FAM-MGB/NFQ
*rpoB*	GCGTTAAGCCTATTGTATCTGCTGTT	AGTAAGCTCAGCCAAAGGATTGAC	CAACCAGTCAGCTTTC	FAM-MGB/NFQ

### DNA pre-amplification

*B*. *burgdorferi*-specific gene targets were enriched by pre-amplification PCR with DNA extracted from 1 mL of sample. Total extracted DNA (3.125 μL) was mixed with 6.25 μL of TaqMan PreAmp Master Mix (Life Technologies, Carlsbad, CA) and 3.125 μL of TaqMan assay pool (obtained by 100-fold dilution of the TaqMan assays with nuclease-free water) for PCR with a protocol of 95°C for 10 min, 14 cycles of 95°C for 15 s and 60°C for 4 min, and a final hold at 4°C. Pre-amplified DNA was diluted five-fold with DNA suspension buffer before use in PCR. In 2016, the extracted DNA was suspended in 12.5 μL of DNA suspension buffer; thus, only one fourth (3.125 μL) of the material was utilized for pre-amplification PCR. The remainder of the procedure remained the same.

### Molecular detection of *B*. *burgdorferi* genes from patient samples by dPCR and qPCR

dPCR was performed on a BioMark platform (Fluidigm Corporation, San Francisco, California) with qdPCR37K integrated fluidic chips, following the manufacturer’s instructions with slight modifications. Instead of using 1.8 μL of DNA template, 2.1 μL of diluted pre-amplified DNA was used in each 6-μL PCR reaction. Patient samples underwent dPCR with each of the four TaqMan assays in a singleplex format using the TaqMan Gene Expression Master Mix (Life Technologies, Carlsbad, California) with a protocol of 50°C for 2 min, 95°C for 10 min, and 40 cycles of 95°C for 15 s and 60°C for 1 min. The presence of a single red spot with a sigmoidal amplification curve with a cycle threshold (Ct) value ≤23 in each panel was considered as a positive result.

qPCR was performed with 3 μL of diluted pre-amplified DNA as the template and the TaqMan Gene Expression Master Mix in a total volume of 20 μL on a QuantStudio 7 Flex Real Time PCR System (Life Technologies, Carlsbad, California), following the vendor’s instructions. The PCR parameters were the same as those described above. A Ct value <35 combined with a sigmoidal amplification curve was considered as a positive detection result. All qPCR reactions were performed in triplicate and repeated thrice to monitor reproducibility.

## Results

### Analytical specificity and sensitivity of *B*. *burgdorferi*-specific TaqMan assays

The *B*. *burgdorferi*-specific TaqMan assays were designed with the aid of bioinformatic tools to ensure maximum specificity for the target organism. *In silico* analysis of the primer and probe sequences with the corresponding sequences from various species in the *B*. *burgdorferi* sensu lato complex (all available annotated sequences) assessed the specificity of our designed assays, particularly for the *B*. *burgdorferi* genome (**S1 Data file**). The *in silico* specificity of the TaqMan assays for the *B*. *burgdorferi* genes (*ospA*, *ospC*, *fla*, *rpoB*) was confirmed by experimental testing with 1 ng each of total DNA from different sources (human, *Treponema denticola*, and 27 clinically relevant microbial species listed in **S2 Table**) by qPCR and then confirmed by dPCR. A pre-amplification step with the TaqMan assays was performed prior to dPCR analysis. Quantitative genomic DNA from *B*. *burgdorferi* (ATCC; Cat No. 35210DQ), *B*. *afzelii* (Vircell; Cat No. MBC078), and *B*. *garinii* (Vircell; Cat No. MBC077) served as controls in this experiment. None of the TaqMan assays showed cross-reactivity with any other pathogenic bacterial or human DNA; only the *fla* gene assay showed some cross-reactivity with *B*. *afzelii* and *B*. *garinii* DNA (**S1 Fig**).

**Fig 1:**
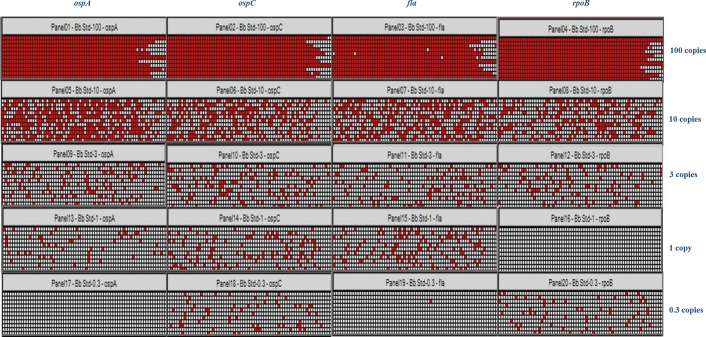
Sensitivity testing of *B*. *burgdorferi*-specific TaqMan assays. Heat map of the gene detection achieved by digital PCR (dPCR). The sensitivity of the TaqMan assays was assessed by diluting the *B*. *burgdorferi* genomic DNA standard (ATCC; Cat No. 35210DQ) in DNA suspension buffer (with poly(A) carrier DNA) and then spiking it with human DNA (1 μg) extracted from healthy control blood specimens. Serial dilutions were performed to obtain 100, 10, 3, 1, and 0.3 genome copies, which were subjected to dPCR with the *ospA*, *ospC*, *fla*, and *rpoB* TaqMan assays. The detection sensitivity was interpreted as three genome copies for the four genes tested, and we considered a gene to be positive whenever a signal was detected in the testing panel. Three genome copies of *Borrelia* were consistently detected in 100% of cases, even though the number of positive partitions/replicates (770 wells in each panel) varied for each test.

The sensitivity of each TaqMan assay for detecting *B*. *burgdorferi* DNA was measured twice, once using blood spiked-in with cultured bacteria (not bacterial DNA; the spiked-in sample underwent DNA extraction and downstream processing before PCR analysis) and once with blood spiked-in with quantitative amounts of *B*. *burgdorferi* genomic DNA. The pre-amplification step was always performed prior to PCR analysis. When analyzing blood spiked-in with cultured bacteria, it was found that all four *B*. *burgdorferi-*specific TaqMan assays were able to detect up to three *Borrelia* genome copies, which was confirmed by repeating the procedure with commercially purchased quantitative DNA (**Fig 1**). Because manually counting spirochetes on a hemocytometer gives approximate and subjective results, we measured the sensitivity of the TaqMan assays again using known amounts of quantitative *B*. *burgdorferi* DNA purchased from the ATCC (Manassas, Virginia). We found that the assay can consistently (100% of the times) detect three genome copies of *B*. *burgdorferi* for all four genes tested in the dPCR format (**Fig 1**). However, the number of positive/negative replicates in each panel (770 wells in each panel) varied when the samples were tested on different occasions. This variance could be due to the qualitative nature of the diagnostic assay, in which the detection sensitivity relies on a non-quantitative pre-amplification step. Our interpretation of the detection sensitivity is based on the ability of the assay to consistently detect all four genes in the dPCR chip despite a varying number of positive/negative replicates in each panel. Due to statistical variations, for tests of one copy or less, we were able to detect *B*. *burgdorferi* DNA in some but not all panels. Similar results were obtained when qPCR was applied to test the sensitivity of the TaqMan assays (**S3 Table**).

### Pre-analytical processing of blood samples

**Fig 2:**
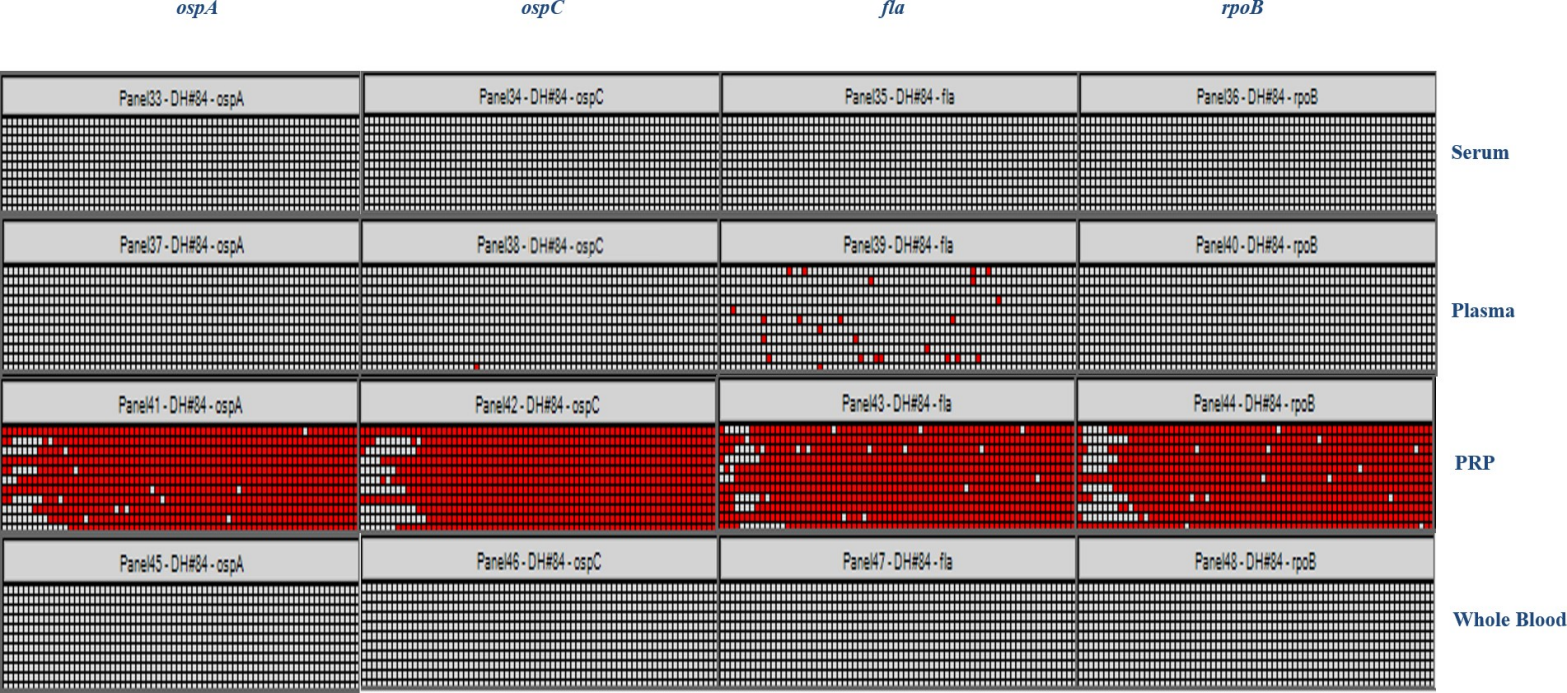
Determination of the optimal sample type based on pre-analytical processing of blood samples. Representative heat map for samples from one of the clinical patients. Clinical blood samples that were found to be positive for Lyme disease (LD) by classical two-tier serology were collected from Danbury Hospital, Connecticut, and processed by different pre-analytical methods to obtain serum, plasma, platelet-rich plasma (PRP), and whole blood (WB) prior to DNA extraction. Following DNA extraction and pre-amplification to enrich for *Borrelia*-specific targets, the samples were subjected to dPCR analysis for the detection of *ospA*, *ospC*, *fla*, and *rpoB* genes using TaqMan assays. The PRP sample was found to give the best detection sensitivity by dPCR for all four *B*. *burgdorferi* genes in the panel.

Pre-analytical processing of blood samples was optimized for *B*. *burgdorferi* detection using serum, plasma, PRP, and WB spiked-in with 3 and 10 copies of cultured *B*. *burgdorferi* (not bacterial DNA). Cultured spirochetes were counted on a hemocytometer, and serial dilutions of spirochetes were performed to achieve the desired concentration for spiking blood samples. The detection sensitivity for the *B*. *burgdorferi* genes was optimal when spiked-in PRP was used as the pre-analytical sample source. Cultured bacteria were first spiked into the different matrices and then subjected to an extraction procedure. The detection rate for the *B*. *burgdorferi* genes was very low when using spiked-in serum, plasma, or WB, even when a pre-amplification step was included before the dPCR step. This result indicates that PRP is the most effective sample type for detecting *B*. *burgdorferi* genes (**S2 Fig**). We utilized clinically diagnosed LD IgM western-blot-positive samples from Danbury Hospital (more than 30 samples) to test this hypothesis and the results confirmed that PRP is the most suitable sample type for detecting LD (**Fig 2**).

For the 2017 patient samples (corresponding to 21 patients), which were collected under our approved IRB protocol and stored at -80°C before use as a PRP pellet instead of PRP, the detection rate of *Borrelia* genes was considerably lower. When the PRP pellets of these 21 patients from three serial visits (excluding the dropouts) were tested by qPCR, only 4 patients showed a significant Ct value for one of the four *B*. *burgdorferi* genes in our panel (**S4 Table**). A Ct value ≤35 was considered as a positive result to eliminate non-specific artifacts from consideration. Hence, the LD detection rate based on PRP pellets for 21 patients was 19.05% after the three visits, which was also confirmed by dPCR. These results indicate that variations in pre-analytical processing and storage methods for different sample types can have a drastic effect on the detection rate of *Borrelia* genes, with PRP being the best sample type for detecting LD by either dPCR or qPCR.

### Evaluation of clinical samples with dPCR technology

**Fig 3:**
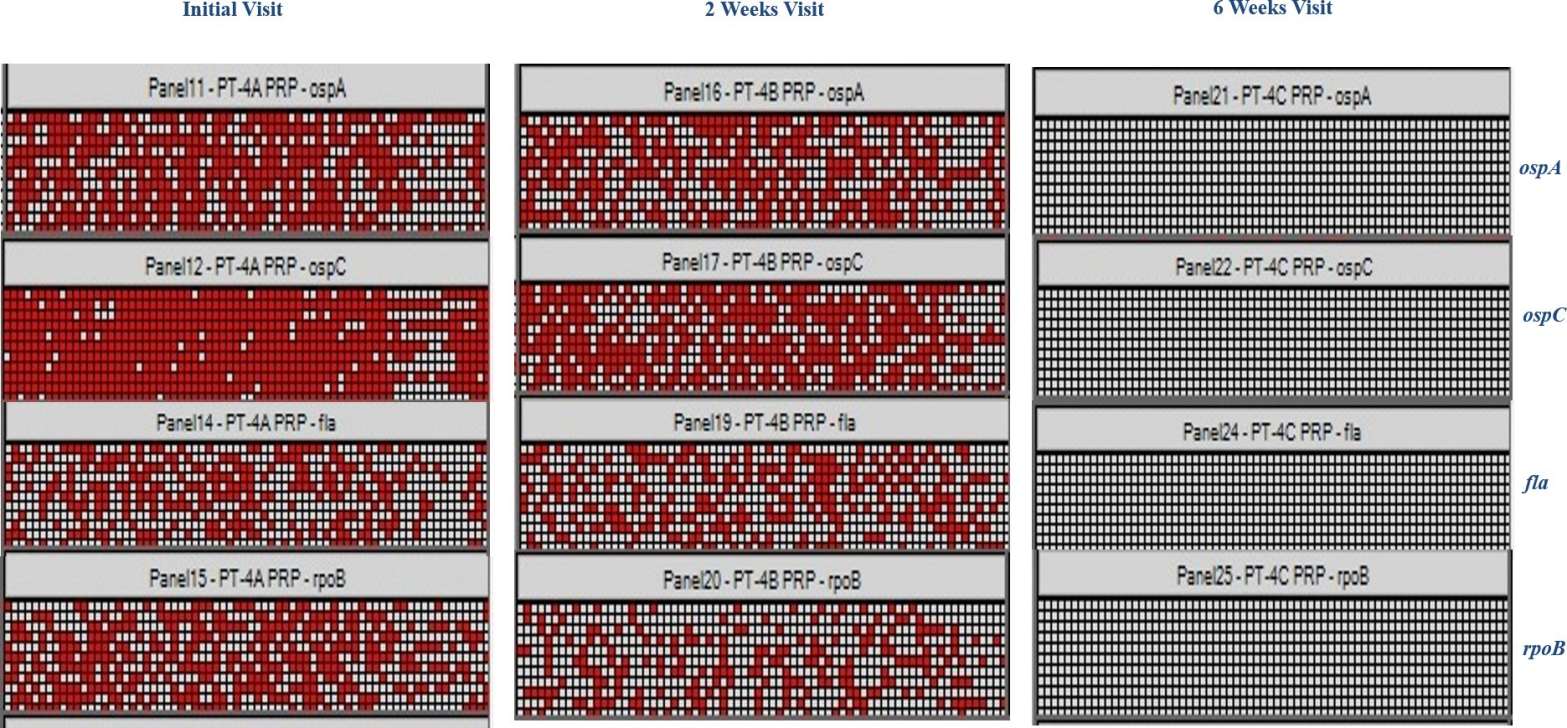
Analysis of Lyme disease (LD) patient samples by digital PCR (dPCR). Representative heat map of dPCR analysis for four *B*. *burgdorferi*-specific genes from a single patient. The visits indicate the duration of antibiotic treatment.

After determining the best sample type and optimizing the assay conditions, the patient samples collected under the approved IRB protocol from 2016 and 2018 were subjected to the dPCR assay to detect the four *B*. *burgdorferi*-specific genes. In 2016, serial blood draws were obtained from 11 patients, of whom 2 dropped out after the initial visit. These patient samples were subjected to pre-analytical processing, DNA extraction, DNA precipitation, and pre-amplification to enrich for *Borrelia*-specific gene targets, followed by the optimized dPCR assay. Only one fourth of the DNA extracted from 1 mL PRP was used in the pre-amplification step and analyzed by dPCR. Among the 11 patients, the dPCR assay could detect *Borrelia* DNA for 7 patients at either the initial or 2-week post-diagnosis visits. A gradual clearing of the signal was observed as the duration of antibiotic treatment increased. A representative heat map for one clinical patient is shown in **Fig 3**.

Among the 2018 samples, 10 out of 14 patients were positive for at least one of the four *B*. *burgdorferi* genes in our panel at their initial visit (*ospA*: 6 patients; *ospC*: 3 patients; *fla*: 4 patients; *rpoB*: 7 patients). In contrast, based on the classical TT testing results, only 2 of these 14 patients were detected as positive for LD by western blot during their initial visit (**S1 Table**). We have included four genes in our *B*. *burgdorferi* detection panel to augment the sensitivity of the diagnostic assay and to ensure proper detection of acute LD patients. In the majority of the patient samples analyzed, not all four genes were detectable by PCR due to varying amounts of bacterial DNA in circulation at the time of testing. Thus, the assay sensitivity was increased by including a panel of four genes, which enables the detection of LD in patient samples that are PCR-positive for one, two, three, or four genes. For the 2018 samples, modifications made to the pre-amplification protocol allowed us to analyze the total DNA extracted from 1 mL of PRP by dPCR (instead of one fourth of the DNA, as applied for the 2016 samples). Among these samples, all enrolled patients were positive for at least three of the four *B*. *burgdorferi* genes in our panel at 2 weeks and 6 weeks post-diagnosis. There was little evidence of signal clearing in these patients as the duration of antibiotic treatment increased.

**Fig 4:**
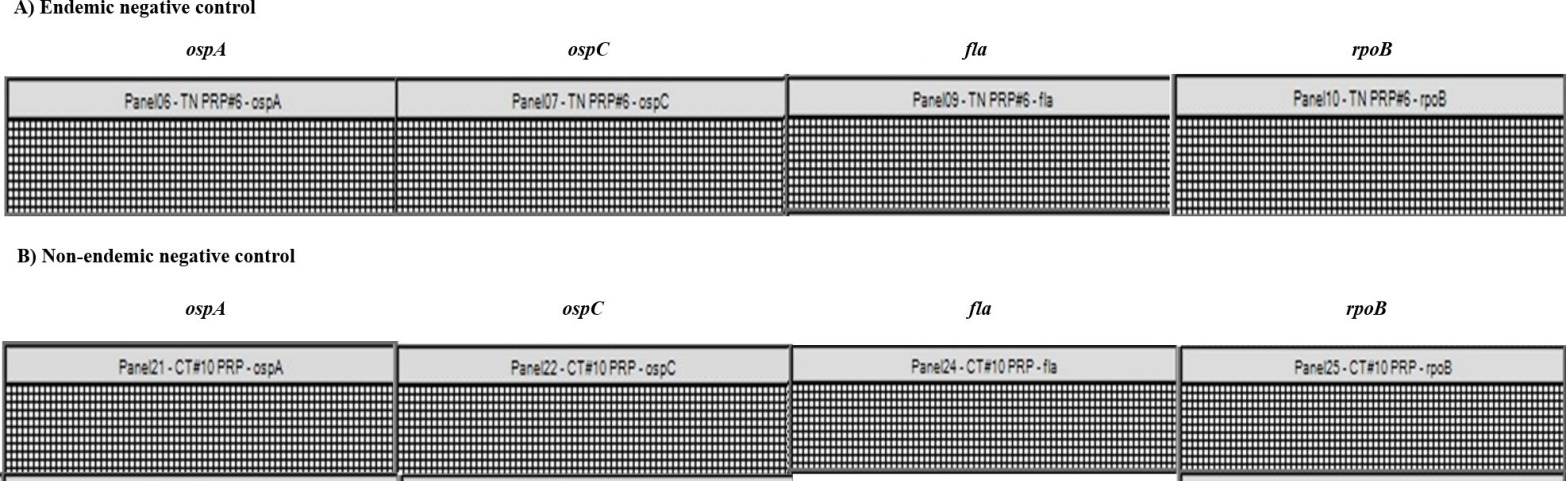
Digital PCR (dPCR) analysis of non-Lyme-disease (LD) control patients. Representative heat map of dPCR analysis from **(A)** an LD-endemic area negative control patient recruited from Connecticut and **(B)** a non-LD-endemic area patient with no LD symptoms from Tennessee. No *B*. *burgdorferi* DNA was detected in samples from the negative control patients.

Compared with results for the 2016 and 2018 patient samples obtained via classical TT testing, the LD detection sensitivity of our dPCR assay was at least two-fold higher. At the initial visit, TT testing showed positive results in only 24.35% of the cases compared with the 58.54% detection rate of the dPCR assay (**Table 2**). In calculating the detection rates, we did not include results for the 2017 samples due to changes in patient sample processing and storage protocols, which had a strong influence on the detection rates of the *B*. *burgdorferi* genes, as mentioned above. *B*. *burgdorferi* DNA was not detected in 100 Connecticut (endemic) or 30 Tennessee (non-endemic) blood samples from patients with no LD symptoms, indicating that our dPCR assay is highly specific for detecting LD. Clinically diagnosed LD patient samples and non-LD controls were processed and tested concurrently. A representative image of the results for the endemic and non-endemic LD negative controls is shown in **Fig 4**.

**Table 2:** Comparison of test results for patients diagnosed with Lyme disease (LD) in this study.

Test Method	Percentage of positive samples (%)		
	At diagnosis	Two weeks Visit	Six weeks Visit
Two-Tiered Serology	24.35	45.84	43.33
Digital PCR	58.54	77.78	83.34

## Discussion

The development of a highly sensitive and specific diagnostic method for detecting LD during acute infection is critical to overcome the ineffectiveness of treatment that arises when LD is detected in later stages. The current CDC-approved TT testing method has a low sensitivity due to a delayed antibody response during the first few weeks of infection and variations in host immune responses. Moreover, the interpretation of TT testing results is subjective, resulting in false-positive and false-negative cases [17,18]. Even the most characteristic symptom of LD, the EM rash, is often either mild or missing, preventing accurate diagnosis [19,20]. This study has demonstrated a much-improved method for detecting *B*. *burgdorferi* directly from WB samples for acute LD cases. Various steps were included to improve the sensitivity, including pre-analytical processing and pre-amplification of *Borrelia-*specific gene targets prior to PCR, and the use of a dPCR platform enabled the development of a robust assay for detecting *B*. *burgdorferi* DNA from patient blood samples. By employing these methods, we overcame the loss of assay sensitivity caused by the very low number of spirochetes in the blood stream of patients [10,13,21,22]. The results from this study indicate that our dPCR assay is successful for cases in which previous assays involving PCR technology have failed to achieve the sensitivity required to become a clinically approved LD diagnostic assay [8,9].

This dPCR assay was designed to have a panel of four gene-specific targets to detect the most common causal agent of LD in the USA, *B*. *burgdorferi*. Although the recently discovered *B*. *mayonii* also causes LD in the USA, it is currently restricted to the upper Midwest region, with few cases reported to date [23]. Among the 18-spirochete species comprising the *B*. *burgdorferi* sensu lato complex, only three cause LD: *B*. *burgdorferi* (North America, Europe), *B*. *afzelii* (Europe, Asia), and *B*. *garinii* (Europe, Asia) [23]. *In silico* alignment of the primer and probe sequences in our diagnostic panel with the corresponding sequences from various species in the *B*. *burgdorferi* sensu lato complex (all available annotated sequences) shows the specificity of our designed assays, especially for the *B*. *burgdorferi* genome (**S1 Data file**). Additionally, this specificity was enhanced by the use of TaqMan chemistry, which adds another level of specificity due to the inclusion of a sequence-specific probe (along with the primers). The TaqMan gene assays in our diagnostic panel were highly specific for detecting *B*. *burgdorferi*. However, the assay for the *fla* gene showed some cross-reactivity with *B*. *afzelii* and *B*. *garinii*, which are restricted to Europe and Asia (**S1 Fig**). The probable reason for this cross-reactivity is the strongly conserved nature of the flagellin gene across the bacterial kingdom.

This study was designed to recruit patients during the initial stages of infection, during the course of antibiotic treatment, and after completion of the antibiotic course. This cohort of patient samples formed the base on which this improved assay was developed. The adoption of dPCR technology achieved the highest sensitivity in detecting *B*. *burgdorferi* DNA and resulted in the detection of as few as three genome copies of *B*. *burgdorferi* (**Fig 1**). This diagnostic assay is qualitative in nature (with a yes/no result for detecting *B*. *burgdorferi* DNA) rather than quantitative, as a pre-amplification step is included to enrich for *B*. *burgdorferi* targets before dPCR analysis. The assay sensitivity was based on the number of times each panel was detected (in our case, three genome copies were detected 100% of the time for all four genes tested). Lower amounts were also detected, but the results were inconsistent with each run. Similar results were obtained when using the qPCR platform (**S3 Table**).

By including a pre-analytical processing step to determine the best sample type for detecting *B*. *burgdorferi* DNA, we have developed a robust assay. This study clearly shows that PRP gives the best detection rates for *B*. *burgdorferi* genes, compared with plasma, serum, and WB (**S2 Fig** and **Fig 2**). The presence of PCR inhibitors, such as hemoglobin, leukocyte DNA, and IgG, may influence the detection of *Borrelia* DNA in WB and plasma [24], and in all likelihood, the spirochetes may co-migrate in the PRP fraction. Recent studies have shown that better results are achieved when larger blood volumes are used in conjunction with other detection methods such as nested PCR [10,13] or isothermal amplification prior to multi-locus PCR/electrospray ionization mass spectrometry [4]. In our study, we used 1 mL of PRP as the starting material to achieve high detection rates for *Borrelia* genes. Depending on the hydration level of the patient, 1 mL of PRP can generally be obtained from 2 mL of WB. This study has also revealed that the storage of the starting material has a substantial impact on the sensitivity of the PCR assay. For the 2017 samples, which were stored at -80°C before use as PRP pellets instead of PRP, the detection rates for *Borrelia* genes were considerably lower (**S4 Table**). LD was detected in only 19.05% of the 21 clinical patients after three serial visits.

The assay sensitivity was increased by modifying the sample processing and pre-amplification steps prior to dPCR, thus overcoming the challenge of low circulating spirochete DNA levels in clinically diagnosed patient samples (**Fig 3**). By concentrating the DNA, we ensured that the maximum possible bacterial DNA amount was analyzed to improve the assay sensitivity. The pre-amplification step was essential to obtaining enhanced sensitivity in our assay, as *B*. *burgdorferi* gene targets were enriched by this step, thus circumventing the scarcity of spirochetal DNA in the circulating blood of patients. Hence, pre-amplification was included in the standard operating procedure to enrich for *Borrelia* DNA when analyzing patient samples.

Due to assay modifications, the pre-amplification steps for the 2016 and 2018 samples differed in the amounts of DNA that were used for analysis. For the 2016 samples, one fourth of the extracted DNA from 1 mL of the patient sample was utilized in the pre-amplification PCR, while the entire extracted DNA was used for the 2018 samples. Despite this difference in the amounts of DNA analyzed from patient samples for these two years, we achieved high detection rates of *Borrelia* genes (**Table 2**). A plausible explanation could be that stringent requirements for patient recruitment in 2016 (recruited patients were required to have a definite EM rash for participation in the study) may have led to the inclusion of patients with a higher bacterial load and less sample variability, explaining why the 2016 samples with lower levels of analyzed DNA had detection results similar to those of the 2018 patient samples (for which all of the extracted DNA was employed in the pre-amplification step). In 2018, patients with or without an EM rash but meeting other criteria, as reported in the material and methods section, were included in the study. We observed a gradual clearing of the signal as the antibiotic treatment continued in the 2016 patient samples, in contrast to the 2018 patient samples. The cause for this difference is unclear; however, in the patient samples, the integrity of the spirochetes is questionable: the spirochetes can be fragmented, form round bodies or blebs, or even be hiding in biofilms [25–27]. This phenomenon could lead to variability in the extraction of spirochetal DNA from such patient samples when using regular DNA extraction kits. Experiments evaluating the extraction efficiencies of spirochete DNA (in its various forms) with modifications in extraction buffers/methods/kits are needed to identify the optimal methods.

A comparison of the dPCR and TT testing results for clinical patient samples revealed that the assay developed in this study can identify patients during the early stages of infection, when the immune response has not yet developed (**Table 2**). At clinical presentation, our assay was at least twice more effective in diagnosis than the CDC-approved classical serology tests. The sensitivity of the dPCR diagnostic assay was also increased by the inclusion of four *B*. *burgdorferi*-specific gene assays. In some cases, we observed patient samples that were positive for all genes in the panel (2016 samples), while at other times, the samples were positive for only some of the genes (2018 samples). A plausible reason behind this phenomenon is the varying bacterial load of different patient samples. When patients with lower bacterial loads were tested, detection sometimes failed due to the lower statistical probability of incorporation of these low-copy-number DNA targets in the PCR reaction. For patients with an optimal bacterial load, all gene targets were detected each time. Thus, it is necessary to include all four genes in our diagnostic panel to have the most sensitive and specific assay for detecting *B*. *burgdorferi* in LD patients. Additionally, the dPCR assay is highly specific, with no false positives, as none of the 130 negative controls were detected as positive (**Fig 4**).

One of the limitations of this work is the small patient pool to which we had access during the course of the study. A power calculation revealed that 75 patients are required to achieve a statistically significant conclusion. Although more patient samples must be evaluated by this assay, the trends observed thus far are promising. If applied to a clinical setting, the approach developed herein can lead to an early and accurate diagnosis of LD, facilitating timely treatment and reducing antibiotic overuse and associated morbidities.

## Supporting information

S1 Fig Analytical specificity testing of *B*. *burgdorferi*-specific TaqMan assays by digital PCR (dPCR). Heat map of the dPCR analysis, showing the detection of the four TaqMan assays with different organisms. Analytical specificity testing of the *ospA*, *ospC*, *fla*, and *rpoB* TaqMan assays was conducted using DNA from various sources by dPCR. Pre-amplification was performed before dPCR analysis. A *B*. *burgdorferi* genomic DNA standard (ATCC; Cat No. 35210DQ) was used as a positive control in this experiment.
(TIF)

S2 Fig Determination of the optimal sample type for detecting Lyme disease (LD) by digital PCR (dPCR). Cultured bacteria were first spiked into the different matrices (whole blood [WB], serum, plasma, and platelet-rich plasma [PRP]). Following DNA extraction and pre-amplification to enrich for *Borrelia*-specific targets, the samples were subjected to dPCR analysis to detect the *ospA*, *ospC*, *fla*, and *rpoB* genes. The PRP sample type was optimal for detecting all four *B*. *burgdorferi* genes in the panel by dPCR.
(TIF)

S1 Table Serology results of Lyme disease (LD) patients by year. (DOCX)

S2 Table List of pathogenic bacteria used for specificity testing of the TaqMan assays. (DOCX)

S3 Table Sensitivity testing of the *B*. *burgdorferi*-specific TaqMan assays by real-time PCR. Average cycle threshold (Ct) values and standard deviation (SD) from three independent experiments (each performed in triplicate). The number of positive replicates versus the total number of replicates is also shown.
(DOCX)

S4 Table Cycle threshold (Ct) values from real-time PCR analysis of the 2017 patient samples. (DOCX)

S1 Data file *In silico* analysis of the primer and probe sequences of the TaqMan assays with various species of the *B*. *burgdorferi* sensu lato complex (accession numbers included) by multiple-sequence alignment. (DOCX)
