# Supplementary figures and images for: Development of a sensitive molecular diagnostic assay for detecting Borrelia burgdorferi DNA from the blood of Lyme disease patients by digital PCR
Source: PLoS One. 2020 Nov 30;15(11):e0235372. doi: 10.1371/journal.pone.0235372 (PMC7703891; doi:10.1371/journal.pone.0235372)

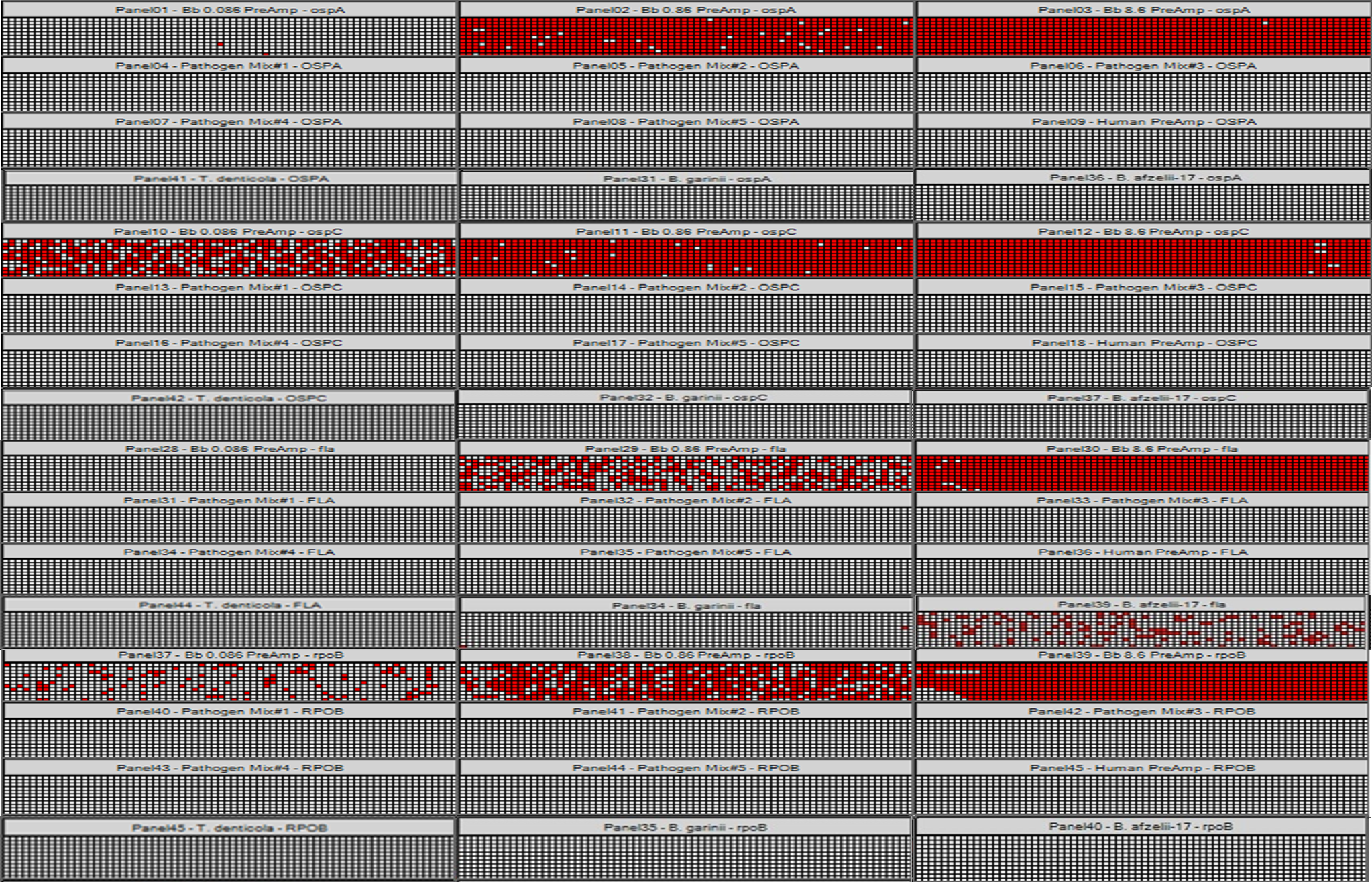

Supplement: S1 Fig — Heat map of the dPCR analysis, showing the detection of the four TaqMan assays with different organisms. Analytical specificity testing of the ospA, ospC, fla, and rpoB TaqMan assays was conducted using DNA from various sources by dPCR. Pre-amplification was performed before dPCR analysis. A B. burgdorferi genomic DNA standard (ATCC; Cat No. 35210DQ) was used as a positive control in this experiment. (TIF) [file pone.0235372.s001.tif]

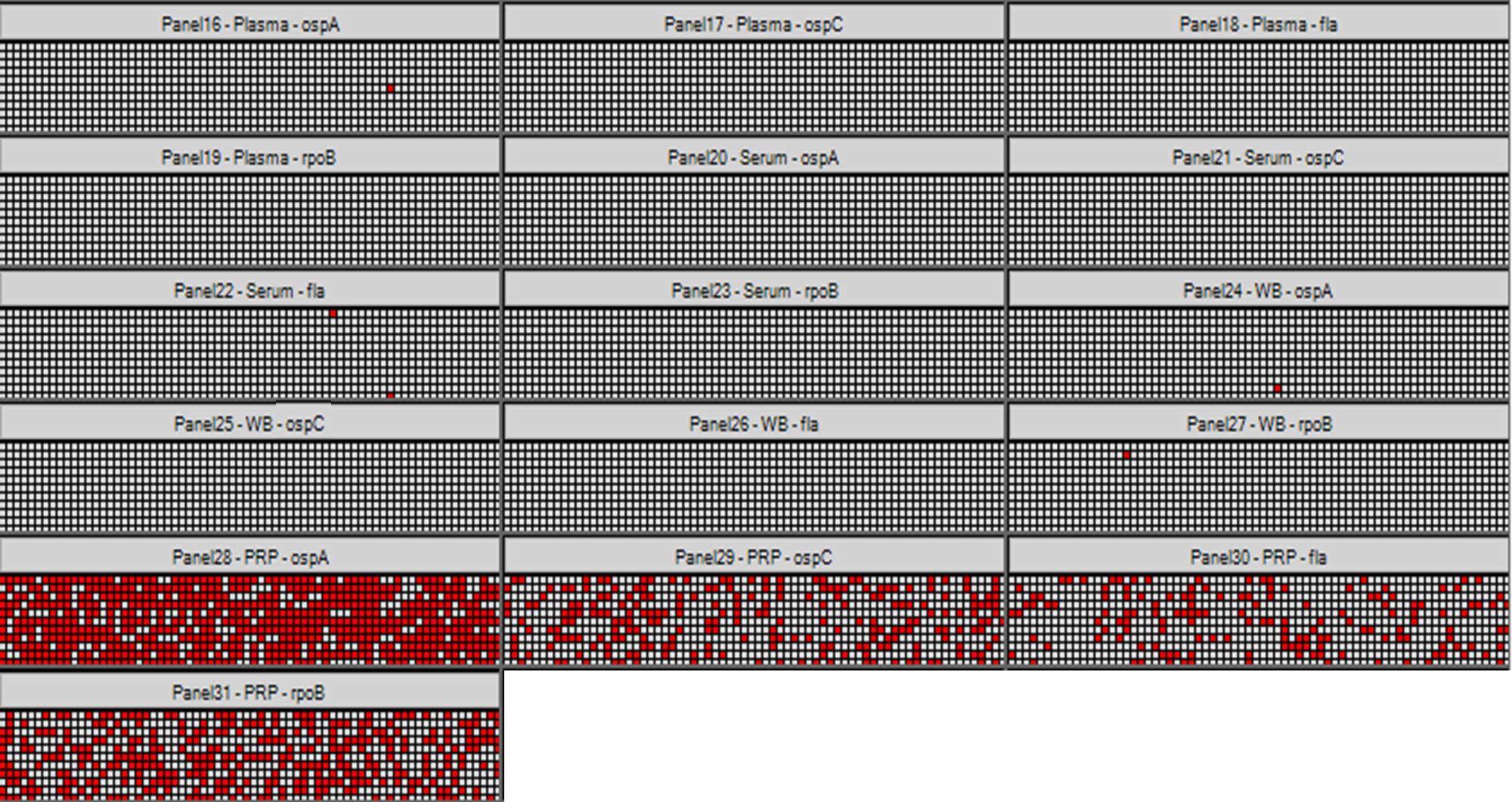

Supplement: S2 Fig — Cultured bacteria were first spiked into the different matrices (whole blood [WB], serum, plasma, and platelet-rich plasma [PRP]). Following DNA extraction and pre-amplification to enrich for Borrelia-specific targets, the samples were subjected to dPCR analysis to detect the ospA, ospC, fla, and rpoB genes. The PRP sample type was optimal for detecting all four B. burgdorferi genes in the panel by dPCR. (TIF) [file pone.0235372.s002.tif]
