# Supplementary material for: Development of a sensitive molecular diagnostic assay for detecting Borrelia burgdorferi DNA from the blood of Lyme disease patients by digital PCR
Source: PLoS One. 2020 Nov 30;15(11):e0235372. doi: 10.1371/journal.pone.0235372 (PMC7703891; doi:10.1371/journal.pone.0235372)
Supplement: S1 Table — (DOCX) [file pone.0235372.s003.docx]

**2016:**

| **Patient ID** | **ELISA^** | **WB* IgM** | **WB* IgG** | **C6 Peptide** |
| --- | --- | --- | --- | --- |
| PT-1A | Negative | Positive | Negative | Negative |
| PT-1B | Dropped out | | | |
| PT-1C | Dropped out | | | |
| PT-2A | Positive | Negative | Negative | Negative |
| PT-2B | Positive | Positive | Positive | Positive |
| PT-2C | Positive | Negative | Negative | Positive |
| PT-3A | Positive | Positive | Negative | Positive |
| PT-3B | Positive | Positive | Negative | Positive |
| PT-3C | Positive | Positive | Negative | Positive |
| PT-4A | Positive | Negative | Negative | Positive |
| PT-4B | Positive | Positive | Negative | Positive |
| PT-4C | Positive | Negative | Positive | Positive |
| PT-5A | Positive | Positive | Negative | Positive |
| PT-5B | Positive | Positive | Negative | Positive |
| PT-5C | Positive | Positive | Negative | Positive |
| PT-6A | Negative | Negative | Negative | Negative |
| PT-6B | Positive | Negative | Negative | Positive |
| PT-6C | Positive | Positive | Negative | Positive |
| PT-7A | Negative | Negative | Negative | Negative |
| PT-7B | Positive | Positive | Negative | Positive |
| PT-7C | Negative | Positive | Negative | Positive |
| PT-8A | Negative | Negative | Negative | Negative |
| PT-8B | Negative | Negative | Negative | Negative |
| PT-8C | Negative | Negative | Negative | Equivocal |
| PT-9A | Negative | Negative | Negative | Positive |
| PT-9B | Dropped out | | | |
| PT-9C | Dropped out | | | |
| PT-10A | Positive | Negative | Negative | Positive |
| PT-10B | Positive | Negative | Negative | Positive |
| PT-10C | Positive | Negative | Negative | Positive |
| PT-11A | Positive | Negative | Positive | Positive |
| PT-11B | Positive | Negative | Positive | Positive |
| PT-11C | Positive | Positive | Positive | Positive |

**2017:**

| **Patient ID** | **ELISA^** | **WB* IgM** | **WB* IgG** | **C6 Peptide** |
| --- | --- | --- | --- | --- |
| PT-1A | Negative | Negative | Negative | Negative |
| PT-1B | Negative | Negative | Negative | Negative |
| PT-1C | Positive | Positive | Negative | Negative |
| PT-2A | Positive | Positive | Positive | Positive |
| PT-2B | Positive | Negative | Positive | Positive |
| PT-2C | Positive | Negative | Positive | Positive |
| PT-3A | Positive | Positive | Negative | Positive |
| PT-3B | Positive | Positive | Negative | Positive |
| PT-3C | Positive | Positive | Positive | Positive |
| PT-4A | Negative | Positive | Negative | Negative |
| PT-4B | Equivocal | Positive | Negative | Negative |
| PT-4C | Positive | Positive | Negative | Negative |
| PT-5A | Positive | Negative | Negative | Positive |
| PT-5B | Positive | Negative | Positive | Positive |
| PT-5C | Dropped out | | | |
| PT-7A | Positive | Positive | Positive | Positive |
| PT-7B | Positive | Positive | Positive | Positive |
| PT-7C | Positive | Positive | Positive | Positive |
| PT-8A | Negative | Negative | Negative | Negative |
| PT-8B | Negative | Positive | Negative | Positive |
| PT-8C | Positive | Negative | Positive | Negative |
| PT-9A | Negative | Negative | Negative | Negative |
| PT-9B | Positive | Negative | Negative | Negative |
| PT-9C | Negative | Positive | Negative | Negative |
| PT-10A | Negative | Positive | Negative | Negative |
| PT-10B | Negative | Positive | Negative | Negative |
| PT-10C | Negative | Negative | Negative | Negative |
| PT-11A | Positive | Positive | Positive | Negative |
| PT-11B | Equivocal | Positive | Negative | Negative |
| PT-11C | Positive | Positive | Negative | Negative |
| PT-12A | Positive | Positive | Negative | Negative |
| PT-12B | Dropped out | | | |
| PT-12C | Dropped out | | | |
| PT-13A | Positive | Positive | Positive | Equivocal |
| PT-13B | Positive | Positive | Positive | Positive |
| PT-13C | Positive | Positive | Positive | Equivocal |
| PT-14A | Positive | Positive | Positive | Positive |
| PT-14B | Dropped out | | | |
| PT-14C | Dropped out | | | |
| PT-15A | Negative | Positive | Negative | Negative |
| PT-15B | Negative | Positive | Negative | Negative |
| PT-15C | Positive | Positive | Negative | Negative |
| PT-16A | Positive | Positive | Negative | Positive |
| PT-16B | Positive | Positive | Positive | Positive |
| PT-16C | Positive | Positive | Positive | Positive |
| PT-17A | Positive | Positive | Positive | Positive |
| PT-17B | Positive | Positive | Positive | Positive |
| PT-17C | Positive | Positive | Positive | Positive |
| PT-18A | Negative | Negative | Negative | Negative |
| PT-18B | Positive | Positive | Negative | Positive |
| PT-18C | Positive | Positive | Negative | Positive |
| PT-19A | Positive | Positive | Negative | Positive |
| PT-19B | Positive | Positive | Positive | Positive |
| PT-20C | Positive | Negative | Positive | Positive |
| PT-20A | Negative | Negative | Negative | Negative |
| PT-20B | Negative | Negative | Negative | Not Available |
| PT-20C | Negative | Negative | Negative | Negative |
| PT-21A | Positive | Positive | Negative | Negative |
| PT-21B | Negative | Positive | Negative | Negative |
| PT-21C | Equivocal | Positive | Negative | Negative |

**2018:**

| **Patient ID** | **ELISA^** | **WB* IgM** | **WB* IgG** | **C6 Peptide** |
| --- | --- | --- | --- | --- |
| PT-1A | Positive | Positive | Positive | Positive |
| PT-1B | Positive | Positive | Positive | Positive |
| PT-1C | Positive | Positive | Negative | Equivocal |
| PT-2A | Negative | Negative | Negative | Negative |
| PT-2B | Negative | Negative | Negative | Negative |
| PT-2C | Negative | Negative | Negative | Negative |
| PT-3A | Negative | Negative | Negative | Negative |
| PT-3B | Negative | Negative | Negative | Positive |
| PT-3C | Negative | Negative | Negative | Negative |
| PT-4A | Negative | Negative | Negative | Negative |
| PT-4B | Dropped out | | | |
| PT-4C | Dropped out | | | |
| PT-5A | Negative | Negative | Negative | Negative |
| PT-5B | Negative | Negative | Negative | Negative |
| PT-5C | Negative | Positive | Negative | Negative |
| PT-6A | Negative | Negative | Negative | Negative |
| PT-6B | Negative | Negative | Negative | Equivocal |
| PT-6C | Negative | Negative | Negative | Negative |
| PT-7A | Negative | Negative | Negative | Negative |
| PT-7B | Negative | Negative | Negative | Negative |
| PT-7C | Negative | Negative | Negative | Negative |
| PT-8A | Negative | Negative | Positive | Negative |
| PT-8B | Negative | Negative | Positive | Negative |
| PT-8C | Negative | Negative | Positive | Negative |
| PT-9A | Negative | Negative | Positive | Positive |
| PT-9B | Negative | Positive | Positive | Positive |
| PT-9C | Dropped out | | | |
| PT-10A | Negative | Positive | Negative | Negative |
| PT-10B | Positive | Positive | Positive | Positive |
| PT-10C | Positive | Positive | Positive | Positive |
| PT-11A | Negative | Positive | Negative | Negative |
| PT-11B | Negative | Negative | Negative | Negative |
| PT-11C | Negative | Negative | Negative | Negative |
| PT-12A | Positive | Positive | Negative | Positive |
| PT-12B | Dropped out | | | |
| PT-12C | Dropped out | | | |
| PT-13A | Negative | Negative | Positive | Negative |
| PT-13B | Negative | Negative | Negative | Negative |
| PT-13C | Negative | Negative | Negative | Negative |
| PT-14A | Negative | Negative | Negative | Negative |
| PT-14B | Positive | Negative | Negative | Negative |
| PT-14C | Dropped out | | | |

Note: ^ELISA- enzyme-linked immunosorbent assay; *WB-western blot; A- Initial visit; B- 2 weeks visit; C- 6 weeks visit.
