# Supplementary material for: Development of a sensitive molecular diagnostic assay for detecting Borrelia burgdorferi DNA from the blood of Lyme disease patients by digital PCR
Source: PLoS One. 2020 Nov 30;15(11):e0235372. doi: 10.1371/journal.pone.0235372 (PMC7703891; doi:10.1371/journal.pone.0235372)
Supplement: S2 Table — (DOCX) [file pone.0235372.s004.docx]

| **Pathogen Mix#1**   1. *Klebsiella oxytoca* (Flugge) Lautrop (ATCC^®^ 49131™) 2. *Staphylococcus aureus* subsp. *aureus* Rosenbach (ATCC^®^ BAA-977™) 3. *Enterococcus faecium* (Orla-Jensen) Schleifer and Kilpper-Balz (ATCC^®^ 700221™) 4. *Staphylococcus aureus* subsp. *aureus* Rosenbach (ATCC^®^ 43300™) 5. *Salmonella enterica* subsp. *enterica* (ATCC^®^ 43300™) |
| --- |
| **Pathogen Mix#2**   1. *Klebsiella pneumoniae* subsp. *pneumoniae* (Schroeter) Trevisan (ATCC^®^ 700603™) 2. *Proteus vulgaris* Hauser emend. Judicial Commission (ATCC^®^ 49132™) 3. *Providencia stuartii* (Buttiaux *et al.*) Ewing (ATCC^®^ 49809™) 4. *Staphylococcus aureus* subsp. *aureus* Rosenbach (ATCC^®^ 29213™) 5. *Shewanella haliotis* (Kim *et al*.) (ATCC^®^ 49138™) |
| **Pathogen Mix#3**   1. *Pseudomonas aeruginosa* (Schroeter) Migula (ATCC^®^ 27853™) 2. *Escherichia coli* (Migula) Castellani and Chalmers (ATCC^®^ 35218™) 3. *Neisseria meningitidis* (Albrecht and Ghon) Murray (ATCC^®^ 13090™) 4. *Streptococcus pneumoniae* (Klein) Chester (ATCC^®^ 49619™) 5. *Moraxella catarrhalis* (Frosch and Kolle) Bovre (ATCC^®^ 8176™) |
| **Pathogen Mix#4**   1. *Radiomyces embreei* Benjamin (ATCC^®^ 13845™) 2. *Streptococcus pyogenes* Rosenbach (ATCC^®^ 19615™) 3. *Staphylococcus aureus* subsp. *aureus* Rosenbach (ATCC^®^ 12598™) 4. *Streptococcus pneumoniae* (Klein) Chester (ATCC^®^ 49136™) |
| **Pathogen Mix#5**   1. *Enterococcus faecalis* (ATCC^®^ 29212^™^) 2. *Pseudomonas putida* (ATCC^®^ 12633^™^) 3. *Ralstonia insidiosa* (ATCC^®^ 49129^™^) 4. *Escherichia coli* (Migula) Castellani and Chalmers (ATCC^®^ 25922™) |
| **Pathogen Mix#6**   1. *Haemophilus influenzae* Blm (+) R-536 2. *Haemophilus influenzae* Blm (-)(ATCC^®^ 19418™) 3. *Haemophilus parainfluenzae* Rivers (ATCC^®^ 9796™) 4. *Neisseria gonorrhoeae* (Zopf) Trevisan (ATCC^®^ 19424™) |
