# Supplementary material for: Development of a sensitive molecular diagnostic assay for detecting Borrelia burgdorferi DNA from the blood of Lyme disease patients by digital PCR
Source: PLoS One. 2020 Nov 30;15(11):e0235372. doi: 10.1371/journal.pone.0235372 (PMC7703891; doi:10.1371/journal.pone.0235372)
Supplement: S3 Table — Average cycle threshold (Ct) values and standard deviation (SD) from three independent experiments (each performed in triplicate). The number of positive replicates versus the total number of replicates is also shown. (DOCX) [file pone.0235372.s005.docx]

| ***B.burgdorferi* DNA copies** | ***ospA*_Mean Ct ± SD (No. of positives)** | ***ospC*_Mean Ct ± SD (No. of positives)** | ***fla*_Mean Ct ± SD (No. of positives)** | ***rpoB*_Mean Ct ± SD (No. of positives)** |
| --- | --- | --- | --- | --- |
| 100 | 22.73 ± 0.13 (9/9) | 22.41 ± 0.02 (9/9) | 21.81 ± 0.05 (9/9) | 22.85 ± 0.12 (9/9) |
| 10 | 26.10 ± 0.04 (9/9) | 25.53 ± 0.04 (9/9) | 25.27 ± 0.07 (9/9) | 26.12 ± 0.09 (9/9) |
| 3 | 28.37 ± 0.04 (9/9) | 27.65 ± 0.03 (9/9) | 26.40 ± 0.05 (9/9) | 27.98 ± 0.15 (9/9) |
| 1 | 30.31 ± 1.53 (7/9) | 30.45 ± 1.92 (6/9) | 27.60 ± 2.12 (6/9) | 30.89 ± 2.15 (7/9) |
| 0.3 | 31.72 ± 0.85 (5/9) | 31.72 ± 1.28 (6/9) | 29.94 ± 0.95 (7/9) | 32.23 ± 1.17 (6/9) |
