# Supplementary material for: Development of a sensitive molecular diagnostic assay for detecting Borrelia burgdorferi DNA from the blood of Lyme disease patients by digital PCR
Source: PLoS One. 2020 Nov 30;15(11):e0235372. doi: 10.1371/journal.pone.0235372 (PMC7703891; doi:10.1371/journal.pone.0235372)
Supplement: S4 Table — (DOCX) [file pone.0235372.s006.docx]

| **Patient ID#** | **Real-time PCR Ct values of PRP Pellets indicating detection** | | | |
| --- | --- | --- | --- | --- |
|  | *ospA* | *fla* | *ospC* | *rpoB* |
| PT-1A | Negative | Negative | Negative | Negative |
| PT-1B | Negative | Negative | Negative | Negative |
| PT-1C | Negative | Negative | Negative | Negative |
| PT-2A | Negative | Negative | Negative | Negative |
| PT-2B | Negative | Negative | Negative | **30.98** |
| PT-2C | Negative | Negative | Negative | Negative |
| PT-3A | Negative | Negative | Negative | Negative |
| PT-3B | Negative | Negative | Negative | Negative |
| PT-3C | Negative | Negative | Negative | Negative |
| PT-4A | Negative | Negative | Negative | Negative |
| PT-4B | Negative | Negative | Negative | Negative |
| PT-4C | Negative | Negative | Negative | Negative |
| PT-5A | Negative | Negative | Negative | Negative |
| PT-5B | Negative | Negative | Negative | Negative |
| PT-5C | Dropped out | | | |
| PT-6A | Negative | Negative | Negative | Negative |
| PT-6B | Dropped out | | | |
| PT-6C | Dropped out | | | |
| PT-7A | Negative | Negative | Negative | Negative |
| PT-7B | Negative | Negative | Negative | Negative |
| PT-7C | Negative | Negative | Negative | Negative |
| PT-8A | Negative | Negative | Negative | Negative |
| PT-8B | Negative | Negative | Negative | Negative |
| PT-8C | Negative | Negative | Negative | Negative |
| PT-9A | Negative | Negative | Negative | Negative |
| PT-9B | Negative | Negative | Negative | Negative |
| PT-9C | Negative | Negative | Negative | Negative |
| PT-10A | Negative | **34.14** | Negative | Negative |
| PT-10B | Negative | **36.1** | Negative | Negative |
| PT-10C | Negative | Negative | Negative | Negative |
| PT-11A | Negative | **28.44** | Negative | Negative |
| PT-11B | Negative | **37.92** | Negative | Negative |
| PT-11C | Negative | Negative | Negative | Negative |
| PT-12A | Negative | Negative | Negative | Negative |
| PT-12B | Dropped out | | | |
| PT-12C | Dropped out | | | |
| PT-13A | Negative | Negative | Negative | Negative |
| PT-13B | Negative | Negative | Negative | Negative |
| PT-13C | Negative | Negative | Negative | Negative |
| PT-14A | Negative | Negative | Negative | Negative |
| PT-14B | Dropped out | | | |
| PT-14C | Dropped out | | | |
| PT-15A | Negative | Negative | Negative | Negative |
| PT-15B | Negative | Negative | Negative | Negative |
| PT-15C | Negative | Negative | Negative | Negative |
| PT-16A | Negative | Negative | Negative | Negative |
| PT-16B | Negative | Negative | Negative | Negative |
| PT-16C | Negative | Negative | Negative | Negative |
| PT-17A | Negative | Negative | Negative | Negative |
| PT-17B | Negative | Negative | Negative | Negative |
| PT-17C | Negative | Negative | Negative | Negative |
| PT-18A | Negative | Negative | Negative | Negative |
| PT-18B | Negative | Negative | Negative | Negative |
| PT-18C | Negative | Negative | Negative | Negative |
| PT-19A | **28.52** | Negative | Negative | **29.02** |
| PT-19B | Negative | Negative | Negative | Negative |
| PT-19C | Negative | Negative | Negative | Negative |
| PT-20A | Negative | Negative | Negative | Negative |
| PT-20B | Negative | Negative | Negative | Negative |
| PT-20C | Negative | Negative | Negative | Negative |
| PT-21A | Negative | Negative | Negative | Negative |
| PT-21B | Negative | Negative | Negative | Negative |
| PT-21C | Negative | Negative | Negative | Negative |

Note: A- Initial visit; B- 2 weeks visit; C- 6 weeks visit.
