# Supplementary material for: Development of a sensitive molecular diagnostic assay for detecting Borrelia burgdorferi DNA from the blood of Lyme disease patients by digital PCR
Source: PLoS One. 2020 Nov 30;15(11):e0235372. doi: 10.1371/journal.pone.0235372 (PMC7703891; doi:10.1371/journal.pone.0235372)
Supplement: S1 Data file — (DOCX) [file pone.0235372.s007.docx]

**Color codes: Forward Primer-Yellow; Reverse Primer-Green; Probe-Blue**

***ospA* Alignment**

Forward primer GGCACTTCAACTTTAACAATTACTGTAA

B.burgdorferi -----------GCTTGGAATTCAGGCACTTCAACTTTAACAATTACTGTAAACAGT----

B.bissettii -------TGGGAAATGGGATGCAGGCACTTCAACTTTAACAATTACTGTAAACAAC----

B.carolinensis ------CTGGAAAATGGGATGCAGGAACTTCAACCTTAACAATTAGCGTAAACAGC----

B.spielmanii ------CTGGCGCATGGGATTCAAAAACTTCTACTTTAACAATTACTGTTAACAGC----

B.garinii ------CTGGAAAATGGGATTCAAAGACTTCCACTTTAACAATTAGTGTGAATAGC----

B.lusitaniae ------CTGGGGCTTGGGATGAAAATACTTCCACTTTAACAATTACTGTTAACAGC----

B.mayonii ------CAGGTAAATGGGAAGACAGTACTAGCACTTTAACAATTAGTGCTGATAGC----

B.afzelii -----GTAAGTTCTAAAGACAAAACATCAACAGATGAAATGTTCAATG-AAAAAGG----

B.americana -----GTAACTTCCAAAGACAAGTCATCAACAGAAGAAAAATTCAATG-AAAAAGG----

B.andersonii -----GTAACTTCCAAGGACAAGTCAACAACAGAAGCAAAATTCAATG-AAAAAGG----

B.valaisiana --AAAGTAAATTTCAAAGACAAGTCTTTCACAGAAGAAAAATTCAATG-AAAAAGG----

B.californiensis -------GGCAACAGTAGACAAGCTTGAGCTTAAAGGAACTTCCGACAAAAACAATGGAT

B.yangtzensis ----GATGGCAACAGTAGACAAAGTTGAGCTTAAAGGAACTTCTGATAAAAACAATGGAT

B.bavariensis ---------TAACAGTAGACAAGCTTGAGCTTAAAGGAACTTCTGATAAAAACAACGGTT

B.japonica CCCTAATGGCAACAGTAGACAAGCTTGAGTTAAAAGGAACTTCTGATAAAAACAACGGTT

* ** * *

Reverse Primer TACAGTACAACAATACG

B.burgdorferi --AAA**AAAACTAAAGACCTTGTGT**TTACAAAAGAAAACACAATTACAGTACAACAATACG

B.bissettii --AAAAAAACTAAAGCCCTTGTATTTACAAAACAAGACACAATTACATCACAAAAATACG

B.carolinensis --AAAAAAACCAAAGACCTTGTATTTACAAAACAAGACACAATTACAGTACAACAATAC-

B.spielmanii --AAAAAAACTAAAGATCTTGTATTTACAAAACAAGACACAATAACTGTACAAAAGTACG

B.garinii --CAAAAAACCAAAAACCTTGTATTCACAAAAGAAGACACAATAACAGTACAAAAATACG

B.lusitaniae --AAAAAAACTAAAGACCTTGTGTTCTTAGCAGATGGCACAATAACTAAACAAAGTTACA

B.mayonii --AAAAAAACCAAAGACTTGGTGTTCTTAACAGACGGTACAATTACAGTACAAAACTATG

B.afzelii -TGAATTGTCTGCAAAAACCATGACAAGAGAAAATGGAACCAAACTTGAATATACAGAAA

B.americana -TGAAGTATCTGAAAAAACAATAACAAGAGCAGACGGAACCAGACTTGAATACACAGAAA

B.andersonii -TGAAGTGTCTGAAAAAACAATGACAAGAGCAAACGGAACTACTCTTGAATACTCACAAA

B.valaisiana -TGAAGTG------------ATGATC----TAGGCGAAACCAAACTTGAA-ACTTTCAAA

B.californiensis CTGGAGTACTTGAAGGCGTAAAAGATGACAAAAGTAAAGTAAAATTAACAGTTTCTGACG

B.yangtzensis CTGGAATGCTTGAAGGCGTGAAAGATGACAAAAGTAAAGTAAAATTAACAATTTCTGATG

B.bavariensis CTGGAACACTTGAAGGTGAAAAAACTGACAAAAGTAAAGTAAAATTAACAATTGCTGATG

B.japonica CTGGAACACTTGAAGGTGCAAAAACTGACAAAAGTAAAGTAAAATTAACAATTTCTGACG

* * * * *

Reverse Primer ACTCAAATGGC

B.burgdorferi ACTCAAATGGCACCAAATTAG----------------------

B.bissettii ACTCAGCAGGAACCAACTTGGAAGG------------------

B.carolinensis -------------------------------------------

B.spielmanii ACTCAGCAGGAACTAATTTAGAGGGCTCTGCAGTCG-------

B.garinii ACTCAGCAGGCACCAATCTAGAAGGCAAAGCAGTCGAAATT--

B.lusitaniae ACACAAACGGGGACAAGCTTGAAGGCCAAGCTGAAGAAGTT--

B.mayonii ACACAGCTGGCACTTCCCTTGAAGGATCACCAAAGGAAATT--

B.afzelii TGAAAAGCGATGGAA---CCGGAAAAGCTAAAGAAGTTTT---

B.americana TTAAAAGCGATGGAT---CCGGAAAAGCTAAAGAGGTTTTAAA

B.andersonii TGACAAATGAAGACAATGCTGCAAAAGCAGTAGAAACTCTT--

B.valaisiana GAAGATGGAACATTAGTGTCA-AGAGTCTG-------------

B.californiensis ATCTAAGCACAACCACACTTGAAGTTTTAA-------------

B.yangtzensis ATCTAAGCAAAACCACACTTGAAACTTTCAAAGAAGATGGT--

B.bavariensis ACCTAAGTCAAACTAAATTTGAAATTTTCAAAGAAGATGGC--

B.japonica ATCTAAGTAAAACCACACTTGAAACTCTAAAAGAAGATGGCA-

***ospC* Alignment**

Forward primer GGTTGAAGCGTTGCTGTCATCTATA

B.burgdorferi --TGAAAGAGGTTGAAGCGTTGCTGTCATCTATAGAT**GAAATTGCTGCTAAAG**CTATTGG

B.bavariensis -GTGAAAGAAGTTGAGGCTTTGCTTTCATCTATAGATGAACTTT---CTAAAGCTATTGG

B.americana TGTAAAAGAAGTTGAGACTTTGCTTTCATCTATAGATGAACTTG---CTAAAGCTATTGG

B.japonica -GTTAAAGAAATTGAGGCTTTACTTTCATCTATAGATGAACTTGCTACTAAAGCTATTGG

B.yangtzensis -GTGAAAGAAGTTGAGACTTTGCTTGCATCTATCGACGAATTTGCTACTAAAGCGGTTGG

B.bissettii -GTAAAAGAAGTTGAAACTCTACTTACATCTATAGACCAACTTGCTACTAAAGCTATTGG

B.carolinensis TGTAAAAGAAGTTGAGGCTTTGCTTTCATCTATAGATGAACTTGCTACTAAAGCTATTGG

B.garinii -GTGAAAGAAGTTGAGGCTTTGCTTTCATCTATAGATGAA------ATTCAAGCTATTGG

B.tanukii -GTGAAAGAAATTGAGACTTT--TTTCATCTATAGATGAACTTGCTACTAAAGCTATTGG

B.spielmanii -GTGAAAGAAGTTGACTTTGC--TTTTATCTATAGATGAACTTGC---TAAAGCTATTGG

B.afzelii -GTTAAAGAAGTTGAGACTTTGGTTTCATCTATAGATGAACTTG---CTAAAGCTATTGG

B.lusitaniae ----------------GCTTTGGTTTTATCTATAGATGAAATTGGTACTAAAGCTATTGG

B.kurtenbachii CGTGAAAGAAGTTGAAACTTTGCTTACATCTATAGATGAACTTG---CTAAAGCTATTGG

B.valaisiana TGTGAAAGAAGTTGAGACTTTGCTTTCATCTATAGATGAACTTG---CTAAAGCTATTGG

B.miyamotoi -GTAAAAGAAGTAGAGACTTTAGTTAAGTCAATAAATGAACTTG---CTAAAGCTATTGG

B.mayonii TGTGAAAGAGGTTGAAGCTTTGGTTGCATCCATAGATGAACTTG---CTAAAGCTATTGG

* ** ** * ** * **** ****

Reverse Primer TACACCAAAATAATGGTTTGGATACCGA

B.burgdorferi TAAAAAAATACACCAAAATAATGGTTTGGATACCGAAAA---TAATCACAATGGATCATT

B.bavariensis TAAAAAAATAAAAAATGATGGTACTTTAGATAACGAAGC---AAATCGAAACGAATCATT

B.americana TAAAAAAATACAAAATAATGGT---TTGGTTAACGAAGC---AAATCAGAACGGCTCATT

B.japonica TAAAAAAATACAAAATAACAATGATTTAACTGCCGAAGC---GAATAAGAATGGATCATT

B.yangtzensis TAAAAAAATA---CAGAATAATGGTTTAGGTAACGAACA---GAATCGTAACGAAGCATT

B.bissettii TAAAAAAATAGATCAAAATAATGGGTTAGCTGACGAACA---GGATAAAAACTCATCATT

B.carolinensis TAAAAAAATAGATGCAAATGGTAGCTTGGTTGCCGATGCAACAGATTTTAACACATCGTT

B.garinii TAAAAAAATAGATGGAAATGGT---TTAACTGCTGATG------ATCACAACAATTCATT

B.tanukii TAAAAAAATAGA--GCAAAACT---TTAGGAGCCTTACA---AGATCACAACGGATCATT

B.spielmanii TAAAAAAATAGA--AAAATGGT---TTAGGCACCGAAGC---GAGTCATAACACCTCATT

B.afzelii TAAAAAAATAGATAACAATAATGGTTTAAGTGCTAATGC---GAATTTAAACACCTCATT

B.lusitaniae TCAAAAAATAGATCAAAATAATGGTTTATCCGCTAACGC---GGATAAAAACACATCATT

B.kurtenbachii CCAAAAAATAGATCAAAATAATGGTTTGGGTGCCGTTGC---AGACCACAATGGAACATT

B.valaisiana TAAAAAAATAGATCAAAATGGTGGTTTAGGTAATGACGC---GAATCATAACGGATCATT

B.miyamotoi AAAGAAAATTAAGTCCGATGGGCAGTTTGATACTGAGTC---AGGTAAAAATGGATCATT

B.mayonii TAAAAAAATACAGCAAAATAATGGGTTGGGGAATGAAGC---AGGTAAGAATGGATCATT

* ***** * **

***fla* Alignment**

Forward Primer TCTAGTGGGTACAGAATTA ATCGAG

B.burgdorferi --------------------------------TCTAGTGGGTACAGAATTA-ATCGAG--

B.carolinensis GTCTCAGGCTTCTTGGACTTTAAGAGTTCATGTTGGAGCAAACCAAGATGA-AGCTA---

B.californiensis GTCTCAGGCTTCTTGGACTTTAAGAGTTCATGTTGGAGCAAATCAAGATGA-AGCTA---

B.americana ----CAAGCTTCTTGGACTTTAAGAGTTCATGTTGGAGCAAACCAAGATGA-AGCTA---

B.andersonii ATCTCAAGCTTCTTGGACCCTAAGAGTTCATGTTGGAGCAAACCAAGATGA-AGCTA---

B.turdi GTCTCAGGCTTCTTGGACCTTAAGAGTTCATGTGGGAGCAAATCAAGATGA-AGCTA---

B.lusitaniae ATCTCAAGCTTCTTGGACTTTGAGAGTTCATGTGGGAGCAAATCAAGATGA-AGCTA---

B.spielmanii AGCTCAAACTTCTTGGACTTTAAGAGTTCATGTGGGAGCAAATCAAGATGA-AGCTA---

B.bissettii ---------------------AGCACCTGCTACAGCGCCTTCTCAGGGTGG-AGTTAATT

B.yangtzensis -----AATCTTAGTAAAACTCAAGAGAAGCTTTCTAGTGGGTACAGAATTA-ATCGAG--

B.tanukii ----------TAGTAAAACTCAAGAGAAGCTTTCTAGTGGGTACAGAATTA-ATCGAG--

B.valaisiana ---------------------AAGAGAAGCTTTCTAGTGGGTACAGAATTA-ATCGAG--

B.garinii ------------------CTCAAGAGAAGCTTTCTAGTGGTTACAGAATTA-ATAGAG--

B.sinica ------------------------AGAAGCTTTCTAGTGGGCACAGAATTA-ATAGAG--

B.mayonii ---------------AAACTCAAGAAAAGCTTTCTAGTGGATACAGAATTA-ATCGAG--

B.miyamotoi ---------------AAACTCAAGAAAAACTTTCTAGTGGGTATAGAATTA-ATCGTG--

B.afzelii ------------TAAAAATTATTAAATTTTTACCGATACGAAATAAAAAAACAAAAGATC

B.bavariensis -----------GAAAGAAACATTTA-CCATTGCATTTACAAAAAAGATAAAAAACTATTT

* *

Forward Primer CTT

Reverse Primer TGGGAGTTTCTGGTAAGATTAATGCTC

B.burgdorferi CTT**CTGATGATGCTGCTGG**CATGGGAGTTTCTGGTAAGATTAATGCTC------------

B.carolinensis TTGCTGTAAATATTTATGCAGCTAATGTTGCAAATCTTTTCTCTGGTGAGG---------

B.californiensis TTGCTGTAAATATTTATGCAGCCAATGTTGCAAATCTTTTCTCTGGTGAGG---------

B.americana TTGCTGTAAATATTTATGCAGCTAATGTTGCAAATCTTTTCTCTGGTGAGGG--------

B.andersonii TTGCTGTAAATATTTATGCAGCTAATGTTGCAAATCTTTTTTCTGGTGAGG---------

B.turdi TTGCTGTAAATATTTATGCAGCTAATGTTGCAAATCTATTTTCTGGTGAGG---------

B.lusitaniae TTGCTGTAAATATTTATGCAGCTAATGTTGCAAATCTGTTTTCTGGTGAGG---------

B.spielmanii TTGCAGTAAATATTTATGCAGCTAATGTTGCAAATCTATTTTCTGGTGAGG---------

B.bissettii CTCCTGTTAATGTTACAACCACAGTTGATGCTAATACATCACTTGCTAAAATAGAAAATG

B.yangtzensis CTTCTGATGATGCTGCTGGTATGGGGGTTTCTGGTAAAATTAATGCTCAAATAAGA----

B.tanukii CTTCTGATGATGCTGCTGGAATGGGGGTTTCTGGTAAAATTAATGCTCAAATAAGA----

B.valaisiana CTTCTGATGATGCTGCTGGTATGGGGGTTTCTGGTAAAATTAATGCTCAAATAAGAGGTT

B.garinii CTTCTGATGATGCTGCTGGTATGGGGGTTTCTGGGAAGATTAATGCTCAAATAAGA----

B.sinica CTTCTGATGATGCTGCTGGTATGGCAGTTTCTGGGAAGATTAATGCTCAAATAAGA----

B.mayonii CTTCTGATGATGCTGCTGGTATGGGAGTTTCTGGTAAGATTAATGCTCAAATAAGAG---

B.miyamotoi CATCTGATGATGCTGCTGGTATGGGTGTTGCTGGTAAGCTTAATTCACAAATTAGA----

B.afzelii CTTTAAAGGATCTTTTGTTAATAATTTGTACTAATTAATTTAAAATTTTATCTAAG----

B.bavariensis TTCAAGAAAATCTTTAA--AATAGAAGAATCTAATAAACTCAAAAAATTAAATGTA----

** * *

***rpoB* Alignment**

Forward Primer GCGTTAAGCCTATTGTATCTG

B.burgdorferi ----------------------GCCTCAAGAATTAATAAGCGTTAAGCCTATTGTATCTG

B.bissettii -------------------------TCAAGAATTAATTAGTGTTAAACCTGTTGTATCTG

B.yangtzensis ------------------------CCCAGGAACTAATAAGTGTTAAACCTATTGTATCTG

B.californiensis ------------------------CCCAGGAACTAATAAGTGTTAAACCTATTGTATCTG

B.valaisiana ---------------------AGCCCCAGGAATTAATAAGCGTTAAACCTATTGTATCTG

B.andersonii -------------------------TCAAGAATTAATAAGCGTTAAACCTATTGTATCTG

B.garinii ----------------------GCCCCAAGAGTTAATAAGCGTTAAACCTATTGTATCTG

B.lusitaniae ---AGGAAGTTTTTAATTTAAAGCCTCAAGAGTTAATAAGCGTTAAACCTATTGTATCTG

B.japonica -------------------------CCAAGAATTAATAAGTGTTAAACCTATTGTATCTG

B.afzelii --AAAGAAGTTTTTAATCTAAAACCTCAAGAATTAATAAGCGTTAAACCCATTGTATCTG

B.turdi ------------------------CTCAAGAATTAATAAGTGTTAAACCTATTGTATCTG

B.spielmanii ----------------------GCCTCAAGAATTAATAAGTGTTAAACCTATTGTATCTG

B.miyamotoi --AAAGAGGTATTTAGTCTTAAACCTCAAGAATTAATAAGTGTTAAACCTATTGTTTCTG

B.mayonii GAAGAATAATCAATAAATATAAATATAAAAGAGCAAAG--TTTTTGCTCTTTTATATTTA

B.bavariensis ---------TCAATAACTGTACCTTCAGTACCATGAGGAACTTTTAAAGAATTATTTTTA

** ** * * *

Forward Primer CTGT-T

B.burgdorferi CTGT-TAAAGAATTTTTTG**CAACCAGTCAGCT**------------**TTC**ACAGTTTATGGAT

B.bissettii CTGT-TAAAGAATTTTTTGCAACCAGTCAGCT------------TTCACAGTTTATGGAC

B.yangtzensis CTGT-TAAAGAATTTTTTGCAACCAGCCAGCT------------TTCGCAGTTCATGGAT

B.californiensis CTGT-TAAAGAATTTTTTGCAACCAGTCAGCT------------TTCACAGTTTATGGAT

B.valaisiana CTGT-TAAGGAATTTTTTGCAACCAGCCAGCT------------TTCGCAGTTCATGGAT

B.andersonii CTGT-TAAAGAATTTTTTGCAACCAGTCAGCT------------TTCACAGTTTATGGAT

B.garinii CTGT-TAAAGAATTTTTTGCAACCAGCCAGCT------------TTCGCAGTTTATGGAT

B.lusitaniae CTGT-TAAAGAATTTTTTGCAACCAGTCAGCT------------TTCGCAGTTTATGGAT

B.japonica CTGT-TAAAGAATTTTTTGCAACCAGTCAGCT------------CTCACAGTTCATGGAT

B.afzelii CTGT-TAAAGAATTTTTTGCAACCAGCCAGCT------------TTCTCAGTTCATGGAT

B.turdi CTGT-TAAAGAATTTTTTGCAACTAGTCAGCT------------TTCGCAGTTCATGGAT

B.spielmanii CTGT-TAAAGAATTTTTTGCAACTAGCCAACT------------TTCGCAGTTTATGGAT

B.miyamotoi CTGT-TAAAGAATTTTTTGCAACTAGTCAGCT------------TTCACAATTTATGGAT

B.mayonii -TATTTATTGATTATTCTTCAAT--TTGAACT---AAGTTT---TTAATAATTAATAATT

B.bavariensis ACATCTTTCGCTTTTTCTCCAAAAATGGAAGTTAAAAGTCTAAATTCAGGAGTAATGTCT

* * * * ** * *** * * * * **

Reverse Primer GTCAATCCTTTGGCTGAGCTTACT

B.burgdorferi CAGGTCAATCCTTTGGCTGAGCTTACTCACAAAAG-------------------

B.bissettii CAGGTCAATCCTTTGGCTGAACTTACTCACAAAAG-------------------

B.yangtzensis CAGGTCAATCCTTTGGCCGAACTTACTCATAAAAGGCGCCTTAATGCTCTTGG-

B.californiensis CAAGTCAATCCTTTAGCCGAACTTACTCATAAAAGGCGTCTTAATGCCCTTGGA

B.valaisiana CAGGTCAATCCTTTGGCCGAACTTACTCATAAAAGGCGTCTTAATGCTCTTGG-

B.andersonii CAGGTCAATCCTTTGGCTGAGCTTACTCACAAAAGGCG----------------

B.garinii CAGGTTAATCCTTTGGCCGAACTTACCCACAAAAGA------------------

B.lusitaniae CAGGTCAATCCTTTGGCCGAACTTACTCATAAAA--------------------

B.japonica CAGGTTAATCCTTTGGCCGAACTTACCCATAA----------------------

B.afzelii CAGGTCAATCCTTTAGCCGAACTTACTCACAAA---------------------

B.turdi CAGGTTAATCCGTTAGCCGAACTTACTCACA-----------------------

B.spielmanii CAAGTTAATCCTTTGGCCGAACTTACTCA-------------------------

B.miyamotoi CAAGTTAATCCCTTGGCTGAATTGACACATAAAAG-------------------

B.mayonii AATTTTAATT-TTTAATTAATTTTGATTATTAAAA-------------------

B.bavariensis CCTTCTGACT-TTGGAGTAACTTTACCAACTAAAATAT----------------

* * * *

| **Accession Numbers** | |  |  |  |  |
| --- | --- | --- | --- | --- | --- |
| **Organisms** |  | ***ospA*** | ***ospC*** | ***rpoB*** | ***fla*** |
| *Borrelia burgdorferi B31* | | AE000790.2 | U01894.1 | AE000783.1 complement (401693..405160) | NC_001318.1 |
| *Borrelia mayonii* | | NZ_CP015795.1 | KR154297.1 | NZ_CP015780.1 complement (402471..405938) | NZ_CP015780.1 complement(148759..149769) |
| *Borrelia miyamotoi* | |  | CP010328.2 | NZ_CP021872.1 complement(400363..403830) | NZ_CP021872.1 complement(147918..148925) |
| *Borrelia afzelii* | | AY597032.1 | AY363711.1 | AF525483.1 | DQ650337.1 |
| *Borrelia bavariensis* | | JX274591.1 | AJ749867.1 | CP028872.1 (499526..502993) | CP028872.1 ( 757402..758412) |
| *Borrelia garinii* | | GU906888.1 | JF331429.1 | CP003151.1 complement (402465..405932) | CP003151.1 complement(147182..148192) |
| *Borrelia japonica* | | Y10893.1 | AB001376.1 | AF164227.1 |  |
| *Borrelia lusitaniae* | | DQ379495.1 | EF179584.1 | AF164228.1 | LC035453.1 |
| *Borrelia sinica* | |  |  |  | AB022138.1 |
| *Borrelia spielmanii* | | DQ133519.1 | CP001467.1 | ABKB02000007.1 complement(8423..11890) | DQ111034.1 |
| *Borrelia tanukii* | |  | AB000353.1 |  | D82848.1 |
| *Borrelia turdi* | |  |  | NZ_QBLM01000001.1 ( 532338..535805) | KT207788.1 |
| *Borrelia valaisiana* | | AB016979.1 | EU135607.1 | NZ_CP009117.1 complement(400642..404109) | NZ_CP009117.1 complement(146850..147860) |
| *Borrelia yangtzensis* | | EU325679.1 | EU135610.1 | NZ_JACHFG010000002.1 (30119..33586) | NZ_JACHFG010000002.1 (285897..286907) |
| *Borrelia americana* | | EU081296.1 | HM146421.1 |  | KX022979.1 |
| *Borrelia andersonii* | | AY654919.1 |  | AF164234.1 | DQ393342.1 |
| *Borrelia californiensis* | | DQ393326.1 |  | NZ_JACHFB010000002.1 (30199..33666) | DQ393348.1 |
| *Borrelia carolinensis* | | EU085402.1 | HM208381.1 |  | KF793050.1 |
| *Borrelia kurtenbachii* | |  | JQ773448.1 |  |  |
| *Borrelia bissettii* | | AF230516.1 | JF791709.1 | AF164233.1 | KF422807.1 |
